# Metagenomic next-generation sequencing enabled diagnosis of *Aspergillus* spondylitis in an immunocompetent patient: a case report and literature review

**DOI:** 10.3389/fmed.2025.1575363

**Published:** 2025-04-30

**Authors:** Zhou Yang, Sirui Zhou, Zhiying Yang, Ping Liu, Shanming Chen, Weijian Zhu

**Affiliations:** ^1^Department of Orthopedics, Lichuan Hchorizon Hexie Hospital, Lichuan, China; ^2^Department of Orthopedics, Tongji Hospital, Tongji Medical College, Huazhong University of Science and Technology, Wuhan, China; ^3^Department of Respiration, Liyuan Hospital, Tongji Medical College, Huazhong University of Science and Technology, Wuhan, China; ^4^Department of Oncology, Zhongshan Hospital of Dalian University, Dalian, China; ^5^Department of Orthopedics, Liyuan Hospital, Tongji Medical College, Huazhong University of Science and Technology, Wuhan, China

**Keywords:** *Aspergillus fumigatus*, *Aspergillus*, infectious spondylitis, immunocompetent, clinical features

## Abstract

**Background:**

*Aspergillus fumigatus* spondylitis is a rare fungal spondylitis that often occurs in immunocompromised patients. This article reports a case of *Aspergillus* spondylitis with specific image signs, which is rarely reported in an immunocompetent patient.

**Case presentation:**

This is a case of L3-4 segmental *Aspergillus* spondylitis diagnosed. The diagnosis was confirmed by intraoperative metagenomic next-generation sequencing (mNGS) testing of the diseased tissue. The patient was treated with voriconazole and underwent surgical debridement and internal fixation with pedicle screws.

**Conclusion:**

The diagnosis of *Aspergillus* spondylitis is often delayed or missed. Doctors should consider *Aspergillus* spondylitis in the differential diagnosis of unexplained low back pain so that appropriate treatment can be administered to prevent spinal cord injury and disability. *Aspergillus* spondylitis usually results in endplate inflammatory response line on fluid or enhancement sequences and a diffuse low signal in the diseased vertebral body on T2-weighted imaging (T2WI). It also results in large paraspinal abscesses, which requires further research to better differentiate between *Aspergillus* spondylitis and tuberculous spondylitis. Prompt diagnosis and treatment can improve the patient’s prognosis.

## 1 Introduction

*Aspergillus fumigatus* spondylitis is exceptionally rare, particularly in immunocompetent individuals without typical risk factors for fungal infections (1). This case highlights the need to consider *Aspergillus* as a potential cause in cases of refractory infections and underscores the critical role of surgical intervention in managing such conditions. Imaging features often include endplate inflammatory response line on fluid-sensitive or contrast-enhanced sequences, diffuse hypointensity in the affected vertebrae on T2WI, and the presence of large paraspinal abscesses, which may serve as distinguishing characteristics of *Aspergillus* spondylitis.

A literature review was conducted via PubMed using the keywords *Aspergillus*, aspergillosis, *Aspergillus fumigatus*, vertebral osteomyelitis, and spondylodiscitis (Table 1). Patients with multiple causes of immunodeficiency were excluded, including those with HIV infection, hematologic malignancies, chronic liver or kidney disease, solid organ transplantation, immunosuppressive chemotherapy, or corticosteroid treatment. Risk factors for *Aspergillus* infection, such as chronic obstructive pulmonary disease (COPD), tuberculosis, trauma- or surgery-associated infection, and intravenous drug use, were included.

**TABLE 1:** Clinical characteristics of *Aspergillus* spondylitis in immunocompetent patients.

Reference	Year	Age/sex	Predisposing conditions	Presentation	Radiology	Diagnosis	Species	Therapy/duration	Outcome
(8) PA	2014	53/M	Spinal block procedures	Motor Weakness Paresthesia	MRI (L2–L3)	Surgery Biopsy	*Aspergillus* spp.	Laminectomy, amphotericin B /30 days	Recovered
(3) PA	2015	20/M	Past pulmonary tuberculosis	Fever Back pain	MRI (T7–T12)	Biopsy histopathological	*A. terreus*	Voriconazole /16 days Caspofungin /40 days	Sequelae
(1) PA	2019	61/M	Lumbar steroid injection	Back pain	MRI (L3–L4)	Surgery Biopsy Histopathological	*A. nidulans*	Lumbar discectomy Caf /6 weeks Voriconazole /7 months Pos	Recovered
(2) PA	2020	43/M	Brucellosis-related vertebral	Back pain	MRI (L5–S1)	Biopsy Enzyme linked immunosorbent assay	*Aspergillus* spp.	Voriconazole/3 months	Recovered
(4) PA	2015	53/M	Pulmonary granulomatous	Fever cough Back pain	Chest CT MRI (L2–L5)	Surgery	*Aspergillus* spp	Voriconazole/3 months	Recovered
(7) PA	2019	71/M	Abdominal stab wound	Back pain Paresthesia	MRI (T11–T12)	Biopsy Enzyme linked immunosorbent assay Histopathological	*A. terreus*	Laminectomy Voriconazole/5 months	Recovered
(5) PA	2022	54/F	None	Back pain	MRI (T11-T12)	Surgery Histopathological	*Aspergillus* spp	Laminectomy Voriconazole /3 months	Recovered
(6) PA	2023	68/M	None	Thoracolumbar back pain	MRI (T12–L2)	Surgery Histopathological	*A. fumigatus*	Isavuconazole /12 months	Recovered
(10) PA	2023	58/F	None	Low back pain	MRI (L4–5)	Surgery Histopathological	*A. terreus*	Voriconazole /6 months	Recovered
(9) PA	2024	63/M	None	Low back pain	MRI (T12-L1)	Surgery Histopathological	*A. fumigatus*	Voriconazole /1 months	Recovered
Our case	2024	27/M	None	Back pain	MRI (L3–L5)	Surgery Histopathological	*A. fumigatus*	Voriconazole /10 weeks	Recovered

A retrospective review identified 11 cases of *Aspergillus* spondylitis in immunocompetent patients over the past decade, including the present case. The mean age of the patients was 52 years, with a male predominance (82%). The most common clinical manifestations included back or neck pain, occasionally accompanied by fever or neurologic dysfunction. Lumbar spine involvement was the most frequent, accounting for 73% of the cases. The overall treatment outcomes were favorable, with 91% of patients achieving complete recovery. However, one case resulted in severe sequelae. Sixty percent of the cases were managed with a combination of antifungal therapy and surgical intervention, with treatment durations ranging from 4 weeks to 12 months (1–10).

## 2 Case report

**FIGURE 1:**
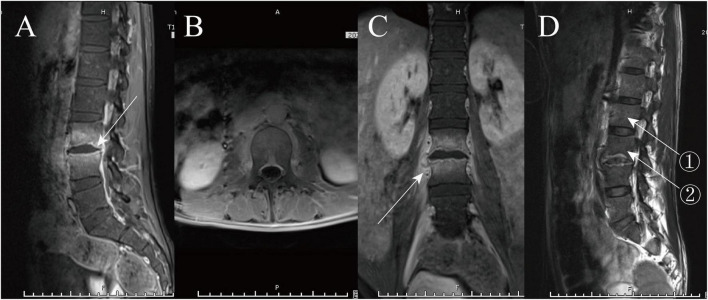
Figure **(A)** shows the enhancement sequence, with the endplate inflammatory reaction line indicated by the white arrow. Figures **(B,C)** display axial and coronal enhancement, respectively, highlighting inflammatory involvement of the surrounding soft tissues. Figure **(D)** shows the T2WI sequence, with density 1 measuring 147 and density 2 measuring 90.

A 27-year-old man presented with low back pain for over a month, which worsened with activity. He had no fever, night sweats, headache, or chest or abdominal pain. His medical history was notable for the absence of immunocompromise and a family history of genetic disorders. T2WI revealed diffuse hypodensity of the L3-4 vertebral body with inflammatory disk erosion, although the disk height remained unchanged. Enhancement sequences demonstrated diffuse hyperdense signals in the L3-4 vertebral body with endplate inflammatory reaction lines (Figure 1).

Physical examination revealed limited lumbar spine motion, positive tenderness over the L3-4 spinous process and paravertebral area, and no neurologic deficits. Laboratory tests showed a white blood cell (WBC) count of 7.09 × 10^9^/L, a neutrophil percentage of 64.9%, a C-reactive protein (CRP) level of 53.1 mg/L, and an erythrocyte sedimentation rate (ESR) of 45 mm/h. All other microbiologic tests were negative.

**FIGURE 2:**
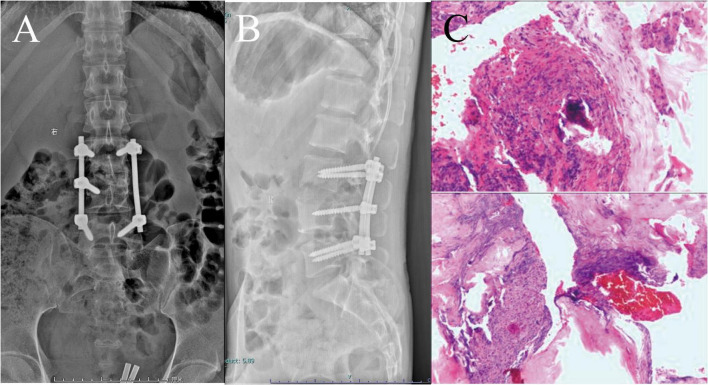
Figures **(A,B)** display the postoperative frontal and lateral plain radiographs, respectively. Figure **(C)** illustrates the histopathological findings of the postoperative vertebral body and disk. The affected intervertebral disk and adjacent vertebral body tissue were predominantly composed of fibrous connective tissue, with localized areas of hemorrhage, necrosis, and scattered neutrophilic infiltration. The magnification of the upper image is 10×, while that of the lower image is 4×.

To alleviate the patient’s pain and reduce inflammation, cefoperazone was administered intravenously at a dose of 7.5 g on the second day after admission. This was subsequently adjusted to 2.5 g intravenously twice daily for 5 days. However, five days later, the patient reported increased low back pain and fever, with a temperature rising to 38.5°C. With the patient’s consent, a posterior lumbar lesion debridement, autologous iliac bone grafting, and pedicle screw fixation were performed to stabilize the spine and remove the lesion (Figure 2).

Postoperative mNGS confirmed *Aspergillus* infection (Supplementary Table 1), whereas Mycobacterium tuberculosis culture results and general bacterial cultures were negative. The treatment regimen was adjusted to intravenous voriconazole, initially at a dose of 300 mg, followed by 300 mg twice daily. After 2 days, the regimen was changed to oral voriconazole at 400 mg twice daily. The patient continued antifungal therapy at home for 1 month. During this period, we conducted blood concentration monitoring of voriconazole in the patient, and the result showed that the blood concentration was maintained at around 2 mg/L.

The patient was followed up for a period of 1 year. At the one month follow-up after the procedure, the symptoms had resolved. Laboratory tests reviewed at this time showed a WBC count of 6.81 × 10^9^/L, a neutrophil percentage of 59.4%, a CRP level of 1.6 mg/L, and an ESR of 6 mm/h. and no recurrence of disease was noted by the end of the 1-year follow-up.

## 3 mNGS detection procedures

In our study, samples were collected following standardized clinical procedures to ensure representativeness and integrity. For instance, fresh infected tissue samples were obtained under sterile conditions in the operating room to minimize contamination. After collection, samples were promptly processed for nucleic acid extraction. Commercial kits were used for nucleic acid extraction, and all steps were performed in accordance with the manufacturer’s instructions to ensure high-quality nucleic acid extraction.

Sequencing libraries were constructed from the extracted nucleic acids through fragmentation, end-repair, and adapter ligation. We used the Illumina NovaSeq platform for high-throughput sequencing. During sequencing, real-time quality control was implemented to monitor data accuracy and reliability. Low-quality sequencing data were filtered out using established bioinformatics tools.

Quality control is a critical component of our mNGS workflow. For sample quality, we ensured that all samples were collected and handled under optimal conditions to prevent degradation and contamination. The purity of nucleic acids was assessed using a NanoDrop spectrophotometer, with acceptable A260/A280 ratios. Library quality was evaluated by agarose gel electrophoresis and Qubit fluorometry to ensure proper concentration and fragment size distribution. For sequencing data quality, we utilized FastQC to assess key metrics such as base quality, sequencing depth, and GC content. Any low-quality reads were removed to ensure high-quality data for downstream analysis.

Our bioinformatics pipeline is designed to provide accurate and comprehensive results. After data preprocessing with Trimmomatic to remove adapter sequences and low-quality bases, the cleaned reads were aligned to reference databases using tools like Bowtie2 and BLAST. This alignment process allows us to identify the presence of various microorganisms, including bacteria, viruses, and fungi. We further analyzed the abundance of each detected microorganism to assess the potential severity of infection.

We established clear criteria for interpreting positive results based on the sample type and clinical context. We set appropriate positivity thresholds accordingly. Moreover, we carefully considered the clinical relevance of positive results by integrating patients’ clinical symptoms, medical history, and laboratory test results. To rule out contamination, especially for low-abundance positives, we traced back every step from sample collection to sequencing.

In the case of our patient, the mNGS detection identified *Aspergillus fumigatus* with a sequence count of 10. This result was interpreted in the context of the patient’s clinical presentation and history. The detection of *Aspergillus fumigatus* that was considered significant given the patient’s clinical symptoms and the potential for invasive fungal infections. The positivity threshold and clinical relevance were carefully evaluated, and the result was communicated to the clinical team to assist in guiding appropriate antifungal therapy.

## 4 Discussion

*Aspergillus* spondylitis is a rare but potentially devastating condition that can occur in both immunocompromised and immunocompetent individuals (11). In this case report, we describe a patient with *Aspergillus* spondylitis diagnosed through mNGS, highlighting the importance of advanced diagnostic techniques in the management of such infections.

mNGS has emerged as a powerful tool for identifying pathogens in complex infections, including spinal infections (12, 13). It allows for the detection of a broad range of microbial pathogens directly from clinical samples, without the need for prior culture or specific amplification. This technique is particularly useful in cases where traditional culture methods have low sensitivity or when the pathogen is difficult to cultivate (14). In our case, mNGS was able to identify *Aspergillus* species in the patient’s tissue samples, despite previous negative results from conventional cultures. This finding underscores the importance of considering mNGS as a diagnostic tool, especially in cases where traditional methods are inconclusive.

The diagnosis of *Aspergillus* spondylitis can be particularly challenging due to its nonspecific clinical and radiological presentations. Patients often present with back pain, fever, and neurological deficits, which can mimic other more common spinal infections such as tuberculosis or pyogenic spondylitis (15). Traditional diagnostic methods, including blood cultures and histopathological examination, may not always yield definitive results, especially in the early stages of the disease (4). In our case, the initial diagnosis was inconclusive, and it was only through mNGS that the presence of *Aspergillus* was confirmed. This highlights the crucial role of mNGS in identifying the causative pathogen, especially when conventional methods fail.

Clinically, our patient presented with progressive back pain and neurological deficits, which are common symptoms in *Aspergillus* spondylitis (3, 10, 15). Imaging studies revealed vertebral destruction and paravertebral inflammatory mass, findings that are consistent with previous reports (5, 10, 11). Interestingly, the enhanced imaging sequence in this case revealed endplate inflammatory reaction lines, findings that align with recent reports by Xu et al. (9) and Jin and Yin (10). Furthermore, T2WI revealed diffuse low signal intensity of the vertebral body (Figure 1), which Xu et al. (9) suggest may result from the paramagnetic material produced by *Aspergillus*. Therefore, large-scale research and investigation are still required to elucidate the specific imaging features of *Aspergillus* spondylitis.

The management of *Aspergillus* spondylitis typically involves a combination of antifungal therapy and surgical intervention. In our case, the patient was treated with voriconazole, which is recommended as the first-line antifungal agent for *Aspergillus* infections (16, 17). Voriconazole has demonstrated efficacy in treating invasive aspergillosis, with a favorable safety profile and good bone penetration (17). Long-term voriconazole therapy can lead to adverse effects such as visual disturbances, hepatotoxicity, and photosensitivity (18). Regular monitoring of liver function, visual assessments, and avoiding excessive sun exposure are essential for managing these risks (18, 19). Additionally, voriconazole has a complex interaction profile, and careful review of concomitant medications is necessary to avoid adverse effects (18, 20). Surgical intervention, including debridement and spinal stabilization, is often necessary to address complications such as spinal instability and neurological compression (1, 6, 7). Our patient underwent surgical debridement and stabilization, which significantly improved their clinical outcome.

Early fungal spondylitis diagnosis and treatment are critical in preventing severe complications such as spinal deformity and permanent neurological damage (Table 2). Delayed diagnosis can lead to the progression of the disease, necessitating more extensive surgical interventions and prolonged antifungal therapy (6, 10). In our case, the use of mNGS facilitated early identification of the pathogen, allowing for prompt initiation of appropriate treatment.

**TABLE 2:** MRI differences between pyogenic spondylitis, tuberculous spondylitis and fungal spondylitis.

Feature	Fungal spondylitis	Pyogenic spondylitis	Tuberculous spondylitis
Location	Lumbar	Lumbar	Thoracic
Number of affected vertebrae	Usually involves two or more connected vertebrae	Usually involves two connected vertebrae	May involve skipping lesions (higher probability)
Degree of vertebral destruction	Mild, often localized erosion	Mild to moderate	Severe, often > 50% destruction
Signal intensity of vertebrae	T2WI typically shows the vertebrae as low signal, while STIR highlights the vertebrae with high signal.	Vertebral high signal on T2WI, vertebral high signal on STIR	Vertebral high signal on T2WI, vertebral high signal on STIR
Paraspinal abscess	Thick, irregular abscess wall	Thick	Thin, regular abscess wall
Vertebral abscess	Rare	Rare	Common
Intradiscal abscess	Rare	Common	Rare

STIR, short tau inversion recovery.

## 5 Conclusion

*Aspergillus* spondylitis is a rare but serious condition that requires a high index of suspicion and advanced diagnostic techniques for early identification. mNGS has proven to be a valuable tool in confirming the diagnosis, especially when traditional methods are inconclusive. A combination of antifungal therapy and surgical intervention is often necessary to achieve optimal outcomes. However, this case report has several limitations. First, it is a single case report, which limits the generalizability of the findings. Second, the long-term outcomes and the impact of voriconazole therapy on patient quality of life require further evaluation. Third, the specific imaging features of *Aspergillus* spondylitis need to be validated in larger cohorts. Future studies should focus on multi-center collaborations to increase sample size, conduct long-term follow-up to assess treatment outcomes, and explore advanced imaging techniques to better differentiate *Aspergillus* spondylitis from other spinal infections.

## Data Availability

The original contributions presented in this study are included in this article/Supplementary material, further inquiries can be directed to the corresponding authors. Gene sequencing has been uploaded into the NCBI database (http://www.ncbi.nlm.nih.gov/bioproject/1202223).
