# Supplementary material for: Metagenomic next-generation sequencing enabled diagnosis of Aspergillus spondylitis in an immunocompetent patient: a case report and literature review
Source: Front Med (Lausanne). 2025 Apr 30;12:1575363. doi: 10.3389/fmed.2025.1575363 (PMC12074920; doi:10.3389/fmed.2025.1575363)
Supplement: Supplementary file 1 [file Table_1.docx]

>P100002760L1C024R00302097899

ATTAGTTATTATTTTATTATTAACATAGGGATAAATAGCTAC

>P100002760L1C030R00400240244

CTCTGCACAGTCATGGACGGCTGCTAGTAGAGTACGAAAATC

>P100002760L1C023R00400093997

AGTAGATTATACTAGGTGACCTACTTACTTCCTGACTTGTGA

>P100002760L1C023R00201134001

AGTGATATTCTCACTGTCTCACTTGCATGCATTCATCTAATT

>P100002760L1C046R00401234381

TTATTCTTAGTTTTCTACTAGTCCTTAATATATTAGGGAATA

>P100002760L1C006R00101230810

AAAAAAGGCAACATCATGCTCCCATATCCTCCATTCACAAAA

>P100002760L1C023R00102458724

AGTTAATCAAACTGTCGCATCTACAACTTCACTTTGAGCCTT

>P100002760L1C030R00200520990

CTGAGGCAGCCAAATTCCATTCTGATCTATCTGACCAGGCTA

>P100002760L1C012R00400447981

ATAGAATCTTGATAAAATATATTCAACTTATACTAGCCTAGT

>P100002760L1C033R00201426109

CTAGTTGCATGGTCTTTCATCCACTTTCTTCCTATTGAGTCC
